# Robot-Assisted Partial Nephrectomy is the Hot Topic in this Number of International Brazilian Journal of Urology

**DOI:** 10.1590/S1677-5538.IBJU.2026.04.01

**Published:** 2026-06-11

**Authors:** Luciano A. Favorito

**Affiliations:** 1 Universidade do Estado do Rio de Janeiro - Uerj Unidade de Pesquisa Urogenital Rio de Janeiro RJ Brasil Unidade de Pesquisa Urogenital - Universidade do Estado do Rio de Janeiro - Uerj, Rio de Janeiro, RJ, Brasil; 2 Hospital Federal da Lagoa Serviço de Urologia Rio de Janeiro RJ Brasil Serviço de Urologia, Hospital Federal da Lagoa, Rio de Janeiro, RJ, Brasil

The July-August number of International Brazilian Journal of Urology presents original contributions with a lot of interesting papers in different fields: Prostate cancer, Robotic Surgery, Kidney cancer, Neonatal circumcision, Telesurgery, Vesicoureteral Reflux, Bladder Cancer, Neuroblastoma, Endourology and Reconstructive Urology. The papers came from many different countries such as Brazil, China, USA, Palestina, Tunisia, France and Germani, and as usual the editor's comment highlights some of them. The editor in chief would like to highlight the work about Robot-assisted Partial Nephrectomy, the cover in this edition.

Dr. Liang and collegues from China, presented in page e20260042 (1) a nice study about the Tubeless Retroperitoneal Robot-assisted Partial Nephrectomy (TLRRPN) and concluded that TLRRPN is a safe, efficient, innovative, cost-effective, and selective surgical procedure. This operation fundamentally reduces the adverse effects associated with tubes in patients and significantly facilitates rapid recovery.

Robotic surgery has revolutionized the surgical treatment of urogenital cancer, resulting in better outcomes and faster recovery (2-9). The present study is interesting and will greatly assist the urologist in diagnosing this disease.

The Editor-in-chief expects everyone to enjoy reading.

## References

[1] Liang H, Xu Y, Peng Y, Huang T, Zheng Q, Yang X (2026). Tubeless Retroperitoneal Robot-assisted Partial Nephrectomy: An Innovative Approach to Treat Early Renal Tumor. Int Braz J Urol.

[2] Moschovas MC, Jaber A, Saikali S, Sandri M, Bhat S, Rogers T (2024). Impacts on functional and oncological outcomes of robotic-assisted radical prostatectomy 10 years after the US Preventive Service Taskforce recommendations against PSA screening. Int Braz J Urol.

[3] Jiao RD, Wang Z, Kong XG, Tang SY, Xia D, Wu ZJ (2025). Clinical outcomes and cost-effectiveness between the Sentire® and da Vinci® systems in robot-assisted radical prostatectomy. Int Braz J Urol.

[4] Gamal A, Moschovas MC, Saikali S, Reddy S, Ozawa Y, Sharma R (2025). Comparing the technological and intraoperative performances of Da Vinci Xi and Da Vinci 5 robotic platforms in patients undergoing robotic-assisted radical prostatectomy. Int Braz J Urol.

[5] Pires RDS, Pereira CWA, Favorito LA (2024). Is the learning curve of the urology resident for conventional radical prostatectomy similar to that of staff initiating robot-assisted radical prostatectomy?. Int Braz J Urol.

[6] Sakthivel DK, Prabhakar P, Raja MJ (2025). Maximal anatomic bladder neck preservation at the prostatic origin (MANO) in robotic radical prostatectomy: does prostate size matter?. J Robot Surg.

[7] Zhang Z, Li Z, Xu W, Wang X, Zhu S, Dong J (2024). Robot-assisted radical nephroureterectomy using the KangDuo Surgical Robot-01 System versus the da Vinci System: a multicenter prospective randomized controlled trial. Int Braz J Urol.

[8] Orsini A, Lasorsa F, Bignante G, Yoon J, Dymanus KA, Cherullo EE (2024). Single port robotic nephrectomy via lower anterior retroperitoneal approach: feasible, safe and effective option in surgically complex patients. Int Braz J Urol.

[9] Silvestri A, Gavi F, Sighinolfi MC, Assumma S, Panio E, Fettucciari D (2025). Management of small renal masses: literature and guidelines review. Int Braz J Urol.

